# ZNF521 Represses Osteoblastic Differentiation in Human Adipose-Derived Stem Cells

**DOI:** 10.3390/ijms19124095

**Published:** 2018-12-18

**Authors:** Emanuela Chiarella, Annamaria Aloisio, Stefania Scicchitano, Valeria Lucchino, Ylenia Montalcini, Olimpio Galasso, Manfredi Greco, Giorgio Gasparini, Maria Mesuraca, Heather M. Bond, Giovanni Morrone

**Affiliations:** 1 Department of Clinical and Experimental Medicine, Laboratory of Molecular Haematopoiesis and Stem Cell Biology, University “Magna Græcia”, Catanzaro 88100, Italy; emanuelachiarella@unicz.it (E.C.); aloisio@unicz.it (A.A.); scicchitano@unicz.it (S.S.); vale.lucchino@studenti.unicz.it (V.L.); ylenia.montalcini@studenti.unicz.it (Y.M.); morrone@unicz.it (G.M.); 2 German Center for Neurodegenerative Diseases (DZNE), Bonn 53127, Germany; valeria.lucchino@dzne.de; 3 Department of Orthopedic & Trauma Surgery, University “Magna Græcia”, Catanzaro 88100, Italy; galasso@unicz.it (O.G.); gasparini@unicz.it (G.G.); 4 Department of Plastic Surgery, University “Magna Græcia”, Catanzaro 88100, Italy; manfredigreco@unicz.it (M.G.)

**Keywords:** zinc finger protein 521 (ZNF521), adipose-derived stem cells (ADSCs), osteogenesis, gene transfer

## Abstract

Human adipose-derived stem cells (hADSCs) are multipotent mesenchymal cells that can differentiate into adipocytes, chondrocytes, and osteocytes. During osteoblastogenesis, the osteoprogenitor cells differentiate into mature osteoblasts and synthesize bone matrix components. Zinc finger protein 521 (ZNF521/Zfp521) is a transcription co-factor implicated in the regulation of hematopoietic, neural, and mesenchymal stem cells, where it has been shown to inhibit adipogenic differentiation. The present study is aimed at determining the effects of ZNF521 on the osteoblastic differentiation of hADSCs to clarify whether it can influence their osteogenic commitment. The enforced expression or silencing of ZNF521 in hADSCs was achieved by lentiviral vector transduction. Cells were cultured in a commercial osteogenic medium for up to 20 days. The ZNF521 enforced expression significantly reduced osteoblast development as assessed by the morphological and molecular criteria, resulting in reduced levels of collagen I, alkaline phosphatase, osterix, osteopontin, and calcium deposits. Conversely, ZNF521 silencing, in response to osteoblastic stimuli, induced a significant increase in early molecular markers of osteogenesis and, at later stages, a remarkable enhancement of matrix mineralization. Together with our previous findings, these results show that ZNF521 inhibits both adipocytic and osteoblastic maturation in hADSCs and suggest that its expression may contribute to maintaining the immature properties of hADSCs.

## 1. Introduction

Mesenchymal stem cells (MSCs) are multipotent adult stem cells that can self-renew and differentiate into osteocytes, chondrocytes, and adipocytes [1,2]. MSCs can be isolated from various tissues, including blood, bone marrow, adipose tissue, cartilage, umbilical cord, dermis, synovium, skeletal muscle, and pericytes [3,4]. Adipose tissue is an abundant and easily accessible source of mesenchymal adult stem cells, and several studies have demonstrated that human adipose-derived stem cells (hADSCs) exhibit adipogenic, chondrogenic, and osteogenic potential [5,6,7,8]. The latter property has stimulated considerable interest for its potential use in emerging clinical applications, especially in orthopedics [9,10].

Bone formation is a process in which osteoprogenitor cells are involved in the synthesis of the bone matrix, deposition, and mineralization and are engaged in bone regeneration by osteoclast regulation [11]. Osteogenic progenitors develop from mesenchymal multipotent cells, whereas the differentiation process towards mature osteoblasts is driven by different mechanical and molecular stimuli such as bone morphogenetic proteins (BMPs) [12], transforming growth factor β (TGF-β) [13], parathyroid hormone (PTH) [14], growth hormone (GH), and insulin-like growth factor-1 (IGF-I) [15]. This process involves the inhibition of the Notch pathway [16] and the Wnt/Ca(2+) pathway. In this case, it was found that miR-26a-5p directly targeted the 3′UTR of Wnt5a [17] and also inhibited the Wnt/β-catenin pathway by Runx2 [18]. The Sonic Hedgehog pathway is instead known to promote osteogenesis [19,20], and mechanical loading has been shown to lead to the activation of the Hh signal through DNA demethylation of the Shh gene promoter in osteogenic differentiation [21].

Osteocytes are considered to be the terminal phase of osteoblast differentiation [22]. They are the most abundant cells of a mature bone and are responsible for its maintenance. Multiple transcription factors drive the control of osteogenesis. Runx2 (Runt-related transcription factor 2) is a key transcriptional regulator during the different stages of osteoblast differentiation together with several other factors, including Twist, Msx2, PLZF, and Osterix (OSX), whereas ATF4 and Zfp521 act as transcription co-factors [23]. Runx2 promotes differentiation by inducing the expression of osteo-specific genes during the early stages of differentiation, whereas for the final stage of mature bone formation, it is thought that its activity needs to be suppressed [23,24]. 

The present study focuses on ZNF521, a multi-zinc finger protein that has been described to antagonize Runx2 activity and modulate both osteogenesis as well as adipogenesis in a variety of cellular models. ZNF521 is a transcription co-factor that is highly expressed in stem/progenitor cells of the hematopoietic [25,26,27,28] and neural systems [29,30,31,32], and implicated in the control of the homeostasis of immature mesenchymal cells [33,34,35,36,37,38,39,40]. 

To more clearly define the role of ZNF521 during osteoblast differentiation, in the light of the multiplicity of molecular signaling pathways involved in this process, a defined system of hADSCs and differentiation medium was used to assess the modulatory effects of ZNF521 in consequence of either enforced overexpression or silencing of its gene. 

## 2. Results

### 2.1. Ectopic Expression of ZNF521 Reduces Osteoblast Differentiation

**Figure 1:**
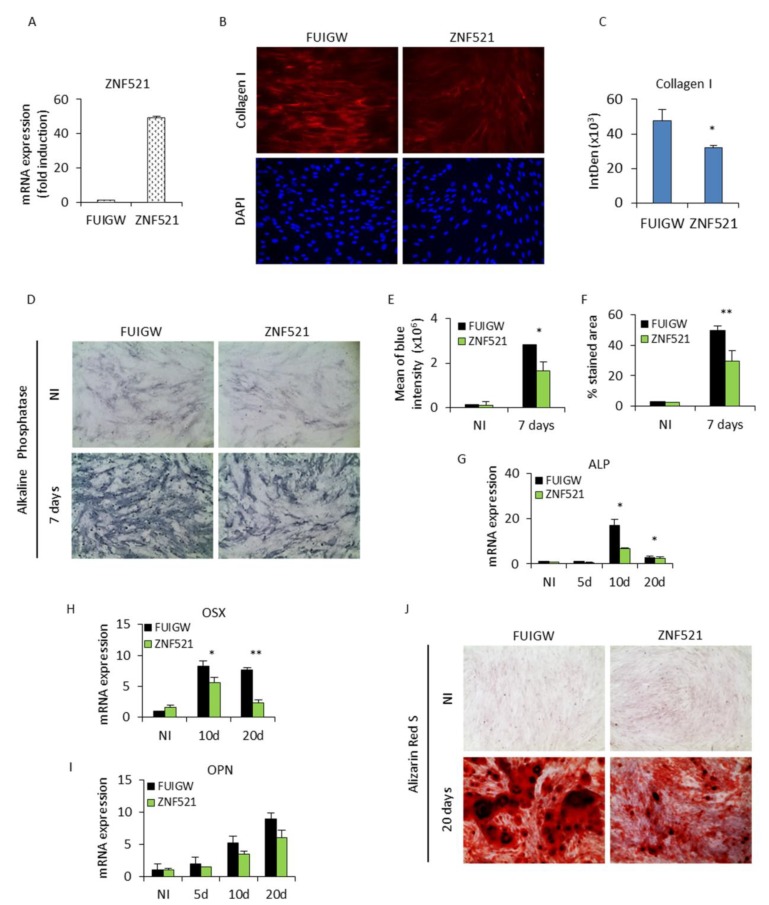
Zinc finger protein 521 (ZNF521) ectopic expression delays osteoblast differentiation in human adipose-derived stem cells (hADSCs). (**A**) ZNF521 overexpression was assessed by Q-RT-PCR to evaluate the fold induction level. (**B**) hADSCs were cultivated in osteogenic induction medium, and after three days the cells were stained with collagen I antibody (red fluorescence) and DAPI (blue) to color the nuclei (magnification 20×). (**C**) Representative immunofluorescence images of osteoblast differentiation are shown and were quantified for fluorescence intensity/cells by ImageJ analysis. (**D**) On day 7, the cells were stained with the BCIP/NBT substrate. Expanded cells demonstrated a basal expression of alkaline phosphatase. The enzymatic activity of alkaline phosphatase (ALP) was markedly decreased in ZNF521 transduced cells compared to the control FUIGW cells (experimental replicate *n* = 2). Representative images are shown with a magnification of 20×. (**E**,**F**) Quantification by ImageJ-based analysis for blue intensity and % area stained by ALP. Q-RT-PCR analysis of (**G**) ALP, (**H**) Osterix (OSX), and (**I**) Osteopontin (OPN) mRNA expression were normalized for the housekeeping gene GAPDH. ZNF521 overexpression resulted in the reduced expression of these osteoblastic markers. (replicate *n* = 2). (**J**) On day 20, Alizarin Red staining was performed to analyze the mineralization process. The accumulation of calcium deposits was significantly reduced in ZNF521-overexpressing cells compared to control cells (experimental replicate *n* = 2). Representative images are shown with a magnification of 20×. Data are represented as means, and error bars denote standard deviation (* *p* < 0.05, ** *p* < 0.005)

To achieve an enhanced overexpression of ZNF521, hADSCs were infected with the lentiviral vector FUIGW-ZNF521 or the control FUIGW empty vector. This resulted in an increase of ZNF521 mRNA levels, quantified by Q-RT-PCR, by at least 40–50-fold (Figure 1A), indicating a typical nuclear expression of the protein [36].

After transduction, these cells were induced for osteoblastic differentiation with a defined commercial osteogenic medium (Life Technologies). On day 3, the cells were examined by immunofluorescence for the expression of collagen type I, which is the most abundant bone matrix protein produced by osteoblasts. Type I collagen expression (Figure 1B) was observed in the form of parallel fiber bundles and, at this time point, was significantly reduced as quantified by ImageJ (Figure 1C) in ZNF521-overexpressing cells compared to cells transduced with the avoid control vector. Alkaline phosphatase (ALP) activity, a characteristic early marker of osteoblast differentiation detected using BCIP/NBT substrate (Figure 1D), showed a significant decrease (Figure 1E,F ImageJ-based analysis) in blue ALP-positive staining after seven days of osteogenic stimulation in the ZNF521-overexpressing hADSCs compared to controls. The cells cultured in osteogenic differentiation medium were also harvested at specific time points for the measurement of ALP mRNA expression (Figure 1G). The ALP expression increased in a time-dependent manner in control FUIGW cells with a peak at 10 days and then decreased, typical of the well-established program of osteoblast differentiation. The peak of induction was significantly reduced in ZNF521-overexpressing cells (Figure 1G). These cells were analyzed for Osterix (OSX), a transcription factor downstream of Runx2 required for osteoblastic differentiation, and Osteopontin (OPN), a protein which plays a role in anchoring the osteoblasts to the mineral matrix of the bone, both of which were reduced upon osteoblastic differentiation with enforced ZNF521 expression (Figure 1H,I). Osteoblasts cultured in osteogenic differentiation medium for three weeks produced extracellular calcium deposits, found in later stages of osteogenesis, which can be specifically stained bright orange-red using the Alizarin Red S dye. The number of mineralized nodules was markedly reduced in ZNF521-overexpressing cells compared to control cells, where large and widely distributed deposits of calcium phosphate were observed (Figure 1J).

These analyses of bone cell-specific markers, collagen I, ALP, OSX, OPN, and calcium deposits indicated that ZNF521 significantly reduced osteogenic differentiation throughout the various stages of osteoblastogenesis in the ADSC model. 

### 2.2. Effect of ZNF521 Knockdown during Osteogenic Differentiation of hADSCs

**Figure 2:**
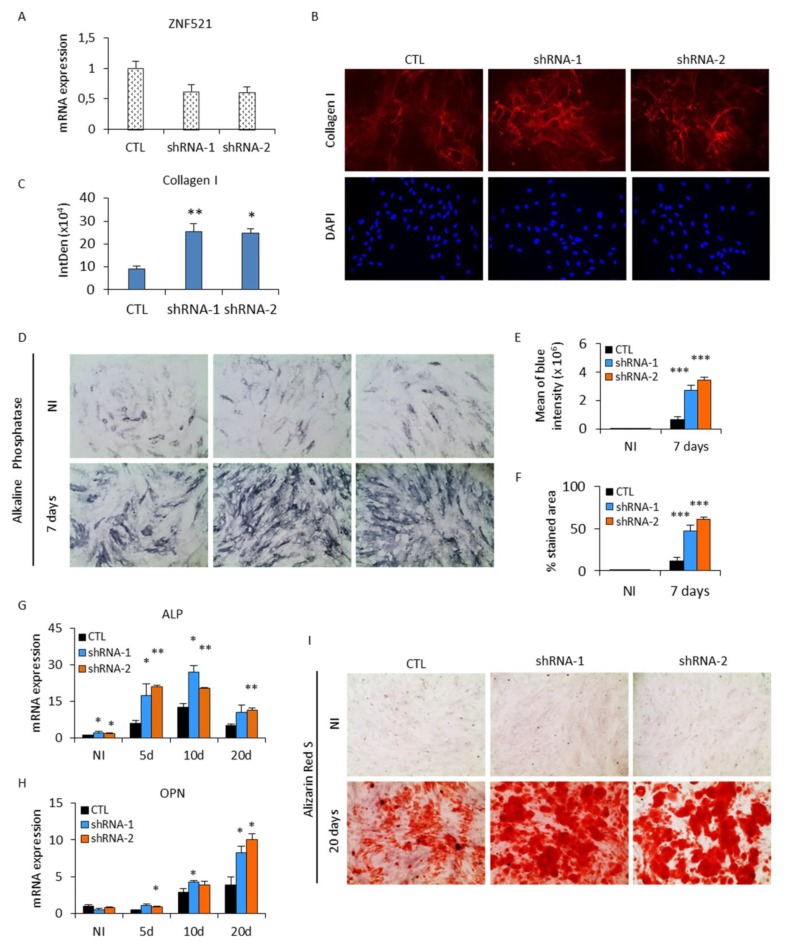
Silencing of ZNF521 promotes human osteoblastic differentiation in hADSCs. (**A**) Quantification of ZNF521 transcript levels by Q-RT-PCR in ZNF521-silenced hADSCs. (**B**) Immunofluorescence staining with anti-collagen I antibody was more pronounced in ZNF521-silenced hADSCs (shRNA-1, -2) compared to control cells on day 3. Representative images are shown with the fibrillar localization of collagen I (red fluorescence) and nuclear staining with DAPI (blue), with a magnification of 20×, (**C**) quantified by ImageJ analysis. (**D**) ZNF521 silencing significantly enhanced ALP activity. On day 7, after BCIP/NBT staining, ZNF521-silenced hADSCs became profoundly dark blue compared to control cells, suggesting that the suppression of this zinc finger protein can accelerate osteoblast formation (magnification 20×) (experimental replicates *n* = 2). (**E**,**F**) Quantification by ImageJ-based analysis for blue intensity and % area stained by ALP. The Q-RT-PCR analysis showed that ZNF521 silencing enhances the expression of the early bone cell-specific marker (**G**) ALP and (**H**) OPN, a later bone matrix protein (experimental replicates *n* = 2). (**I**) The late phase of osteoblast differentiation was analyzed by Alizarin Red S staining. The red color indicates the reaction between calcium ions and Alizarin Red dye and is particularly widespread in ZNF521-silenced cells compared to control cells. This assay was performed twice (magnification 20×). Data are represented as means +SD (* *p* < 0.05, ** *p* < 0.005, *** *p* < 0.0005).

To confirm the ability of ZNF521 in modulating the osteogenic differentiation mechanism, a complementary strategy based on shRNA-mediated gene silencing was used. In hADSCs, ZNF521 expression was reduced using two specific shRNA lentiviral vectors (shRNA-1 and shRNA-2). These have previously been proven to be effective in silencing ZNF521 expression [27,30,36,41]. Q-RT-PCR analysis showed that both shRNAs gave a 50% reduction in ZNF521 mRNA levels in transduced cells (Figure 2A). 

ZNF521-silenced hADSCs were cultured in osteoblastic differentiation conditions. We then evaluated whether ZNF521 knockdown causes an inversion of the trend observed with ZNF521 overexpression. The results showed that the two hADSC populations with ZNF521 knockdown were preferentially induced towards osteoblast differentiation. After three days of osteogenic induction, collagen I staining (Figure 2B) displayed a notably higher degree of staining intensity (Figure 2C, ImageJ quantification) and pericellular organization in silenced hADSCs compared to the control cells. Consistently, the BCIP/NBT staining (Figure 2D), indicating ALP activity in both shRNA-1 and shRNA-2, was considerably stronger than that of the control cells after seven days of differentiation (Figure 2E,F, ImageJ-based method). Q-RT-PCR analysis (Figure 2G) confirmed the result that the transcript levels of ALP showed a higher expression peak in the middle stage of differentiation followed by a typical decrease in its later stages. OPN mRNA also showed a consistent increase in osteogenesis with ZNF521 silencing (Figure 2H). On day 20, Alizarin Red staining (Figure 2I) was also performed to detect calcium deposits. The distribution of these deposits was more widespread and abundant in ZNF521-silenced cells compared to the control counterpart, suggesting that ZNF521 knockdown enhances the late bone mineralization process.

These results indicate that a reduction in ZNF521 facilitates the molecular processes underlying osteogenesis.

## 3. Discussion

ZNF521 has been extensively studied to understand its role in bone formation using a variety of osteoblast differentiation models *in vitro*, as well as in transgenic and knockout mice. In cellular models of osteoblast differentiation, Zfp521 has been shown to bind and antagonize Runx2, repressing its transcriptional activity in a dose-dependent manner [37]. In transgenic mice, where Zfp521 expression was driven by the bone-specific osteocalcin promoter, mature bone formation was enhanced [37].

Additionally, in mice models, Zfp521 has further been shown to control bone mass formation interacting with HDAC3 and reducing Runx2 activity at both the early and late stages of *in vivo* osteoblast maturation [42], thus contributing to the regulation of skeletal development and bone mass. Zfp521 has also been implicated in suppressing Runx2 activity and preventing the hypertrophic conversion of growth plate chondrocytes [43]. 

In a study of human bone marrow-derived mesenchymal stem cells (bmMSCs), Tseng et al. [35] reported that overexpression of Zfp521 repressed osteoblastic differentiation whereas adipogenesis was enhanced. Similarly, in rat mesenchymal cells, the introduction of Zfp521 inhibited osteoblast differentiation whereas the silencing of Zfp521 promoted the expression of osteoblastic markers such as ALP, BSP, OCN, and Runx2, in a process that appeared to be associated with suppression of the Wnt/β-Catenin pathway [40].

In mesenchymal cells from Zfp521-conditional KO mice, Zfp521 has been proposed to repress Zfp423 and maintain bone homeostasis by facilitating BMP-induced osteoblastogenesis at the expense of adipogenic lineage commitment [33]. ZNF521 is known to interact with and repress the activity of EBF1 in the specification of the B-cell lineage [25,27,44,45]. In mesenchymal cells, EBF1 has been proposed to play a key role in promoting adipogenesis by activating PPARγ, the transcription factor that governs adipocyte programming [46,47], and, in turn, has a negative effect on osteogenesis [48]. A tight interplay between Ebf1 and Zfp521 was highlighted by Kiviranta et al. [39], where the concerted cross-talk of the two factors regulates bone homeostasis by controlling the generation of both osteoblasts and osteoclasts. 

It is evident from the above considerations that the cell fate choice of mesenchymal stem cells is governed by a complex network of interactions among multiple signaling pathways (e.g., BMP, Wnt, Hedgehog) and regulatory molecules such as EBF1, RUNX2, and ZFP423, whose activity ZNF521 is known to influence in a synergistic or antagonistic manner. 

In previous studies [36], we demonstrated that ZNF521 efficiently inhibits the adipogenic differentiation of hADSCs in vitro. The findings illustrated in the present paper indicate that, intriguingly, enforced expression of ZNF521 is also able to repress the osteogenic differentiation of the same cells in culture. In contrast, ZNF521 knockdown promotes osteoblastic maturation, as attested by a clear increase in the accumulation of early osteoblastic-related markers and late mineralized bone nodules. Together, these results suggest that ZNF521 may contribute to maintaining mesenchymal progenitors in an immature state by delaying or blocking both adipocyte and osteoblastic differentiation. In future studies, it will be interesting to pinpoint the exact role of ZNF521 and the relevance of its molecular interactions in the control of MSC lineage fate choice. In addition to this, one implication of potential translational relevance derives from the observation that ZNF521 silencing in hADSCs strongly enhances their differentiation toward both adipocytes and osteocytes in response to appropriate stimuli. This may be exploited to achieve the production of large amounts of homogeneously mature adipocytes or osteocytes for biomedical applications through siRNA-mediated silencing of ZNF521, followed by culture in defined conditions.

## 4. Materials and Methods

### 4.1. Cell Culture and Transduction

STEM PRO^®^ human adipose-derived stem cells (hADSCs) were purchased from Life Technologies (Monza, Italy) (cat.no. R7788-115) and grown in MesenPRO RS medium (cat. No. R7788-115 Life Technologies) at 37 °C in a humidified atmosphere containing 5% CO_2_. Cells were infected with two rounds of spin-inoculation using lentiviral vectors [49]. The transduction efficiency was between 60 and 80% for overexpression and over 90% for shRNA silencing (shRNA-1 and -2). The ZNF521 protein expression was evaluated by Western blotting, as documented in Reference [36].

### 4.2. Osteoblastic Differentiation

To induce osteocyte differentiation, 5 × 10^3^ cells/cm^2^ were incubated for 20 days with STEM PRO^®^ osteogenesis differentiation medium (cat. No. A1007201 Life Technologies). Control cells were cultured in MesenPro RS^TM^ medium (Life Technologies). Cells were maintained at 37 °C in a 5% CO_2_ incubator, and the medium was changed every three days. Following seven days of stimulation, the cells were stained with BCIP/NBT substrate. On day 20, Alizarin Red S staining was performed to assess mineralization.

### 4.3. Alkaline Phosphatase Staining

To assess ALP activity, cells were washed twice in PBS, stained with BCIP/NBT Blue Liquid Substrate solution (B3804 Sigma-Aldrich), and incubated in the dark for 10 min at room temperature. The colorimetric reaction was blocked by washing twice with distilled water [50]. The intensity of ALP staining was assessed under an Evos bright-field microscope (EVOS M5000 Cell Imaging System, Life Technologies). The images were quantified by an ImageJ-based method [36] to evaluate blue staining in the RGB 24-bit acquisition. The number of blue pixels with higher intensity than the red or green pixels was divided by the total pixels in the entire field for each of the three independent replicates.

### 4.4. Alizarin Red S staining

hADSCs were cultured in osteogenic differentiation medium for 20 days, and then mineralization was assayed by Alizarin Red S (A5533 Sigma-Aldrich, Milan, Italy) staining. Cells were fixed in 10% formaldehyde solution for 20 min at room temperature. After washing twice with PBS, cells were treated with Alizarin Red solution (2%, pH 4.2) for 40 min and then washed three times with distilled deionized water. Calcium deposits were imaged using an Evos microscope (EVOS M5000 Cell Imaging System, Life Technologies, Monza, Italy). 

### 4.5. Immunofluorescence

Immunofluorescence was performed as described in Reference [51]. hADSCs were washed three times with PBS and fixed with 4% paraformaldehyde for 20 min at 4 °C. Cells were then permeabilized with 0.3% Triton X-100 for 5 min and rinsed three times with cold PBS before being incubated with blocking buffer (PBS, 10% BSA (Albumin Fraction V A1391 Applichem, Milan, Italy), 0.2% Tween-20) for 40 min at room temperature. Rabbit anti-collagen I (ab34710 Abcam, Milan, Italy) primary antibody was diluted 1:500 in blocking buffer and cells were incubated with the antibody overnight at 4 °C. The cells were then washed with cold PBS three times, and incubated with Alexa Fluor 594-labeled anti-rabbit secondary antibody (Life Technologies) diluted 1:800 in blocking buffer (PBS, 2% BSA, 0.2% Tween-20) at room temperature for 1 h. Nuclei were stained with 10 ng/mL DAPI in PBS for 5 min. Cells were visualized, and images were captured with a DFC 3000 G camera mounted on a Leica microscope (DM IL LED, Leica Microsystems Srl, Milan, Italy) at 20× magnification. Staining was quantified as integrated fluorescent density by ImageJ analysis.

### 4.6. Q-RT-PCR

cDNA was synthesized from 1 μg RNA, extracted using TRIzol (Life Technologies) and controlled by NanoDrop quantification, using SuperScript III (Life Technologies). Cycling conditions of 95 °C for 3 min, followed by 45 cycles of 95 °C (10 s), 60 °C (10 s), and 72 °C (10 s), were performed and analyzed on a Bio-Rad iQ5 multicolor detection system (Bio-Rad, Milan, Italy). Analysis of relative gene expression was calculated as 2^−dd*Ct*^. Primers used for Q-RT-PCR were: GAPDH: forward 5′-CACCATCTTCCAGGAGCGAG-3′ and reverse 5′-TCACGCCACAGTTTCCCGGA-3′; ZNF521: forward 5’-TGGGATATTCAGGTTCATGTTG-3′ and reverse 5′-ACTGGAGTTTGGCAGGAGAG-3′; ALP: forward 5′-TAA GGACATCGCCTACCAGC-3′ and reverse 5′-TGGCTTTCTCGT CACTCTCA-3′; OSX forward 5′-CCCAGGCAACACCCTACTC-3′ and reverse 5′-GGCTGGATTAAGGGGAGCAAA-3′ OPN forward 5′-CTCCATTGACTCGAACGACTC-3′ and reverse 5′-CAGGTCTGCGAAACTTCTTAGAT-3′.

### 4.7. Statistical Analysis

Each experiment was performed at least three times unless otherwise specified and two replicates for each sample were used for statistical analysis. In Q-RT-PCR figures, bar graphs represent means and error bars represent standard deviation (SD). Student’s *t*-test was used for statistical analyses (* *p* < 0.05 was considered statistically significant).

## Abbreviations

hADSCs	Human adipose-derived stem cells
ZNF521	Zinc Finger Protein 521
MSC	Mesenchymal stem cells
BMP	Bone morphogenetic protein
TGF-β	Transforming growth factor-beta
PTH	Parathyroid hormone
GH	Growth hormone
IGF-1	Insulin-like growth factor-1
Wnt	Wingless-type MMTV integration site family member
Runx2	Runt-related transcription factor 2
Shh	Sonic Hedgehog
Msx2	Msh homeobox 2
PLZF	Promyelocytic Leukemia zinc-finger protein
Osx	Osterix
ATF4	Activating transcription factor 4
ALP	Alkaline phosphatase
BCIP/NBT	5-Bromo-4-chloro-3-indolyl phosphate /nitro blue tetrazolium
OPN	Osteopontin
HDAC3	Histone deacetylase 3
bmMSCs	Bone marrow-derived Mesenchymal Stem Cell
BSP	Bone sialoprotein
OCN	Osteocalcin
EBF1	Early B-cell factor 1
PPARγ	Peroxisome Proliferator-Activated Receptor gamma
GAPDH	Glyceraldehyde-3-Phosphate Dehydrogenase
